# Cation‐Loaded Porous Mg^2+^‐Zeolite Layer Direct Dendrite‐Free Deposition toward Long‐Life Lithium Metal Anodes

**DOI:** 10.1002/advs.202308939

**Published:** 2024-04-10

**Authors:** Ben Su, Xingyu Wang, Lei Chai, Sida Huo, Jingyi Qiu, Qiang Huang, Shuang Li, Yue Wang, Wendong Xue

**Affiliations:** ^1^ School of Materials Science and Engineering University of Science and Technology Beijing Beijing 100083 China; ^2^ School of Microelectronics Dalian University of Technology Dalian Liaoning 116024 China; ^3^ Research Institute of Chemical Defense Beijing 100191 China; ^4^ School of Materials Science and Engineering Nanjing University of Science and Technology Nanjing Jiangsu 210094 China

**Keywords:** dendrites, Li metal anodes, Mg^2+^‐zeolite, multifunction, plating/stripping behavior

## Abstract

Lithium metal, with ultrahigh theoretical specific capacity, is considered as an ideal anode material for the lithium‐ion batteries. However, its practical application is severely plagued by the uncontrolled formation of dendritic Li. Here, a cation‐loaded porous Mg^2+^‐Zeolite layer is proposed to enable the dendrite‐free deposition on the surface of Li metal anode. The skeleton channels of zeolite provide the low coordinated Li^+^‐solvation groups, leading to the faster desolvation process at the interface. Meanwhile, anions‐involved solvation sheath induces a stable, inorganic‐rich SEI, contributing to the uniform Li^+^ flux through the interface. Furthermore, the co‐deposition of sustained release Mg^2+^ realizes a new faster migration pathway, which proactively facilitates the uniform diffusion of Li on the lithium substrate. The synergistic modulation of these kinetic processes facilitates the homogeneous Li plating/stripping behavior. Based on this synergistic mechanism, the high‐efficiency deposition with cyclic longevity exceeding 2100 h is observed in the symmetric Li/Li cell with Mg^2+^‐Zeolite modified anode at 1 mA cm^−2^. The pouch cell matched with LiFePO_4_ cathode fulfills a capacity retention of 88.4% after 100 cycles at a severe current density of 1 C charge/discharge. This synergistic protective mechanism can give new guidance for realizing the safe and high‐performance Li metal batteries.

## Graphical abstract

A cation‐loaded porous Mg^2+^‐Zeolite layer is proposed to enable the dendrite‐free deposition on the surface of Li metal anode. The stable channels of zeolite provide the low coordinated Li^+^‐solvation groups. Furthermore, the sustained release of loaded Mg^2+^ ensures the uniform diffusion of Li on the lithium substrate during the repeated charging/discharging. The synergistic modulation facilitates the homogeneous Li plating/stripping behavior.

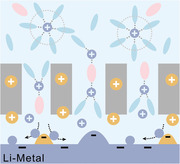

## 1 Introduction

With an ultrahigh theoretical specific capacity of 3860 mAh g^−1^, a small weight density (0.53 g cm^−3^), and the lowest redox potential (≈3.04 V, vs standard hydrogen electrode),^[^ ^1^ ^]^ lithium metal anode has been subjected to extensive attention in recent years. Based on the content of 95% and active material loading of 1.5 g cm^−3^, the Li‐loading capacity of 38 µm graphite active material film can be easily reached by lithium metal anode with a thickness of only 10 µm. Furthermore, the mass of lithium anode is only one‐tenth of that of graphite.^[^ ^2^ ^]^ Lithium metal, widely regarded as the ideal anode materials for the next generation, is the key to breaking through bottleneck of energy density and volume density for lithium‐ion batteries.^[^ ^3^ ^]^ Unfortunately, the practical application of Li metal as anode has so far been hindered by an inevitable mountain: the needle‐like or moss‐like plating of Li (dendritic lithium).^[^ ^4^ ^]^ The generation/growth of Li dendrite brings a series of problems, such as the low coulombic efficiency during the Li plating/stripping process, the short life, the infinite volume expansion, and the crucial safety concerns.^[^ ^5^ ^]^ On the other hand, the non‐aqueous electrolyte components are highly susceptible to be reduced by the highly reactive lithium metal and gradually evolve into the solid electrolyte interface (SEI). This naturally derived SEI is compositionally inhomogeneous, which inevitably produces the inhomogeneous ion transport and further exacerbates the generation of lithium dendrites.^[^ ^6^ ^]^

To realize the dendrite‐free Li deposition during the charging process, various strategies have been proposed in recent years, such as the designs of artificial SEI^[^ ^7^ ^]^ the formation of Li alloys,^[^ ^8^ ^]^ the introduction of lithophilic phases.^[^ ^9^ ^]^ It is established that the high‐concentration electrolytes can induce a stable, anion‐derived SEI due to the low‐coordinated solvated structure.^[^ ^10^ ^]^ This inorganic‐rich SEI achieves a uniform interfacial Li^+^ flux, leading to the elimination of undesired dendritic nuclei. In addition, Metal cations, like Al^3+ [^ ^11^ ^]^ and Cu^2+^,^[^ ^12^ ^]^ can react with Li metal to form a conductive M‐Li alloy phase, which is instrumental in the fast electron transport and uniform charge distribution on the lithium metal substrate, thereby contributing to the uniform Li plating/stripping behavior. Similarly, the in‐suit reduction of lithiophilic matrix on the lithium surface, for instance In^3+ [^ ^9b^ ^]^ and Sn^2+^,^[^ ^13^ ^]^ can guide the homogeneous Li deposition. In regulating lithium deposition, these methods basically depend a single function, which do not simultaneously realize the fast ions transport dynamics at the interface and the regulation of deposition process on the lithium substrate. The relatively single protective function cannot comprehensively meet the practical problems faced by lithium metal anodes. The zeolite molecular sieves have been applied with great success in field of gas separation and chemical catalysis. Owning to the channels with strictly defined size and the excellent electrochemical stability, zeolite molecular sieves have received much attention as electrolyte modifiers in LIBs.^[^ ^14^ ^]^ Meanwhile, the zeolite skeleton structure can be loaded with functional metal ions to realize its multifunctionality without the change of original structure.

Herein, the cation‐loaded Mg^2+^‐Zeolite was proposed to construct lithium metal protective layer. This Mg^2+^‐Zeolite film achieves the dendrite‐free deposition on the surface of lithium metal anode through the combination of desolvation kinetics regulating, SEI optimizing, and electrodeposition mode modulating. Specifically, the highly aggregated solvation structures are trapped in the zeolite pores since the size effect, which contributes to a faster desolvation process and a more stable, ion‐conductive SEI. Furthermore, the co‐deposition of sustained release Mg^2+^ gives a faster migration pathway, leading to the uniform diffusion of Li on the lithium substrate. The synergistic regulation of ion transport at the interface and diffusion kinetics on the substrates facilitate a homogeneous plating/stripping behavior. With such a collaborative mechanism, the high‐efficiency deposition with cyclic longevity exceeding 2100 h is observed in the symmetric Li/Li cell with Mg^2+^‐Zeolite modified anode at a current density of 1 mA cm^−2^. Even at the deposition capacity of 6 mAh cm^−2^, the modified Li anode still maintains a stable cycle >750 h. This multifunctional artificial layer is expected to enable safe and long‐life lithium anodes, providing a new strategy for the practical application of lithium metal batteries.

## 2 Results and Discussion

Benefit from the size‐effect, the well‐defined narrow channels within the Mg^2+^‐Zeolite layer provided Li^+^‐solvated groups with low coordination number (CN). Specifically, the solvent molecules tend to wrap around Li^+^ and form the solvated sheaths due to the strong interaction of oxygen/cation.^[^ ^6^ ^]^ The ion‐exchange between cathode and anode is realized through the transmission of the Li^+^‐solvated sheaths, rather than bare Li^+^. Typically, the solvent molecule/cation coordination ratio decreases with the increasing of the salt concentration in the electrolyte.^[^ ^10a^ ^]^ In addition, anions are involved in the formation of solvated sheath as the CN decreases. Li^+^‐solvated groups with different CN have different sizes, thus the different solvation structures can be screened by the channels with fixed size within Mg^2+^‐Zeolite. This variation of the Li^+^‐solvated structures within the Mg^2+^‐Zeolite channels was well confirmed in Raman spectroscopy and FTIR spectroscopy.

**Figure 1:**
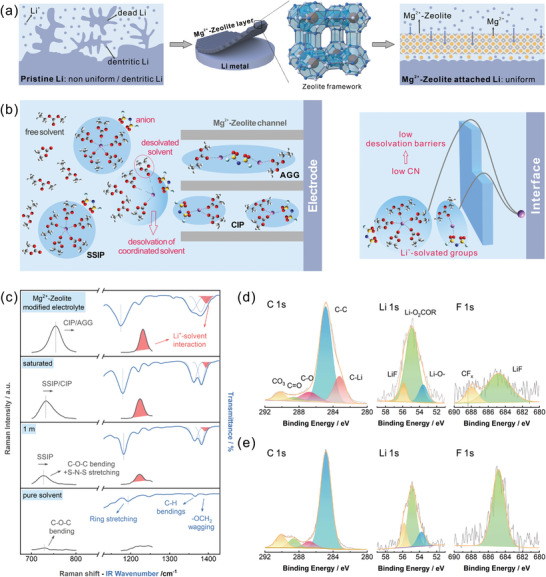
a) Schematic of the Mg^2+^‐Zeolite layer and the diagram of the corresponding effect. b) Schematic of the configuration for the unique Li^+^‐solvated groups inside Mg^2+^‐Zeolite channels. c) Raman and FTIR spectra of various electrolyte. XPS spectra of d) pristine Li metal and e) Mg^2+^‐Zeolite layer modified Li after 10 h of cycling.

For pure DOL+DME solvent, the Raman shifts located at 726 and 1228 cm^−1^ could be attributed to the bending and the stretching vibrations of the C‐O‐C, while the FTIR peaks related to the C‐H bending vibration and ‐OCH_2_ rocking vibration appeared between 1360 and 1390 cm^−1^ (**Figure** **1**c).^[^ ^15^ ^]^ With the addition of LiFSI, the C‐O‐C bending and the S‐N‐S (FSI^−^) stretching combine into one broad Raman shift, which is a notable feature of the binding interactions between anion and Li^+^.^[^ ^16^ ^]^ The peak increased and blue‐shifted with increasing concentration of salt, indicating the enhancement of the ion‐association interactions between Li^+^ and FSI^−^. It is generally accepted that the ion‐association interaction is enhanced with the shift from the solvent‐separated ion pair (SSIP), the contact ion pair (CIP) to the aggregate (AGG) coordination forms.^[^ ^10c^ ^]^ Typically, the AGG peak has a well‐defined position of ≈747 cm^−1^.^[^ ^17^ ^]^ Compared to the saturated electrolyte, the Mg^2+^‐Zeolite modified electrolyte showed the obviously stronger S‐N‐S peak at ≈751 cm^−1^. That is, the DOL+DME‐LiFSI trapped within the Mg^2+^‐Zeolite channels exhibited a more aggregated state, thereby realizing a Li^+^‐solvated groups dominated by the SIP/AGG forms. In this case, there were almost no free solvent molecules in channels, as evidenced by the virtually invisibility of the peaks associated with ‐CH stretching vibrations (Figure S3, Supporting Information). Moreover, the clear peaks associated with Li^+^‐solvent interactions^[^ ^18^ ^]^ could be detected in both Raman and FTIR spectra (highlighted in red, Raman: 1228 cm^−1^; FTIR: 1396 cm^−1^) after introducing LiFSI into the solvent. Besides, these peaks of electrolyte within the Mg^2+^‐Zeolite were clearly stronger than that of saturated electrolyte. Similarly, the ring stretching vibration associated with DOL delivered a slight redshift at ≈1180 cm^−1^ due to the interaction with LiFSI. In summary, the ion‐pair interaction between DOL+DME solvent, Li^+^, and TFSI^−^ became much stronger. Dynamically, the low coordinated Li^+^‐solvated groups captured by the skeleton channels have the lower desolvation barriers.^[^ ^19^ ^]^ Thus, the Mg^2+^‐Zeolite layer achieved a faster desolvation kinetics of Li^+^ at the interface (Figure 1b).

The determination of the SEI composition after cycling was explored by the X‐ray photoelectron spectroscopy (XPS). In the C 1s spectra, the C‐C (284.4 eV), C‐O (286.7 eV), C═O (288.5 eV), and CO_3_ (290.1 eV) associated with typical SEI compositions were all recognized, e.g., LiCO_2_OR, Li_2_CO_3_ (Figure 1d,e).^[^ ^20^ ^]^ An additional peak (283.3 eV) corresponding to the solvent‐induced C‐Li emerged in C 1s spectra of pristine Li foil.^[^ ^21^ ^]^ Similarly, as the typical organolithium compositions, the corresponding Li‐O_2_COR peak (54.9 eV) exhibited the stronger intensity in the blank Li (Figure 1d).^[^ ^9a^ ^]^ In addition, the presence of CF_x_‐related peak (688 eV) also confirmed the substantial existence of organic components. It is deduced that the SEI formed on the pristine Li was mainly composed of organic chain segments, which are easily dissolved in the electrolyte and have no stabilization effect for the Li metal. In contrast, the peaks associated with inorganic components on the surfaces of Li with Mg^2+^‐zeolite layer, like CO_3_ (290.1 eV), LiF (55.9, 684.9 eV),^[^ ^22^ ^]^ all displayed the greater intensity. The enhanced LiF peak substantiated the greater contribution of FSI^−^ within the channels to the evolvement of SEI. This artificial screened CIP/AGG solvation structure promoted the formation of a stable, inorganic‐rich SEI, which have a strong ion transport capacity. Overall, the Mg^2+^‐Zeolite layer provided a faster desolvation process at the interface and a solid electrolyte interface that is easier for ions to cross, leading to the faster Li transport kinetics and the elimination of random dendrite nuclei.

**Figure 2:**
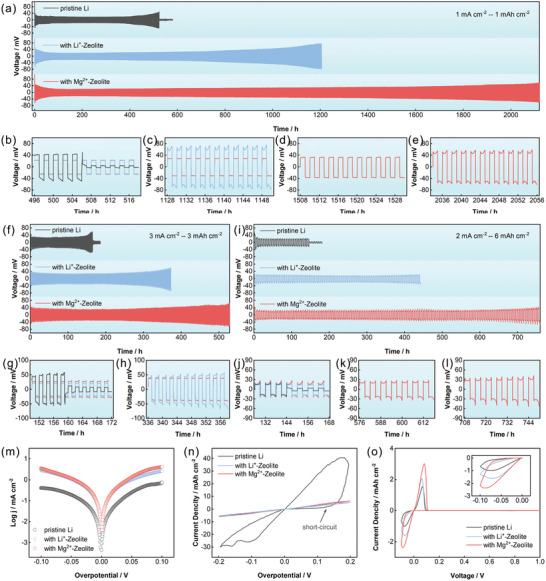
The Li plating/stripping voltage curves of symmetric Li/Li cells with pristine Li and Li^+^/Mg^2+^‐Zeolite artificial layers at current density of a) 1 mA cm^−2^ (with capacity of 1 mAh cm^−2^), f) 3 mA cm^−2^ (with capacity of 3 mAh cm^−2^), and i) 2 mA cm^−2^ (with capacity of 6 mAh cm^−2^), respectively. The corresponding voltage profiles of (a) at b) 249–259, c) 565–575, d) 755–765, and e) 1018–1028 cycles; the corresponding voltage profiles of (f) at g)76–86 and h)169–179 cycles; the corresponding voltage profiles of (i) at j) 22–28, k) 97–103, and l) 119–125 cycles. m) Tafel profiles of the symmetric Li/Li cell with pristine Li and Li^+^/Mg^2+^‐Zeolite modified Li. The CV profiles of n) symmetric Li/Li cell and o) asymmetric SS/Li cell with pristine Li and Li^+^/Mg^2+^‐Zeolite modified Li.

To evaluate the effect of Mg^2+^‐Zeolite layer on long‐term cyclic stability of Li metal anode, the plating/stripping behavior of Li was monitored via symmetric Li/Li cell at the current density of 1 mA cm^−2^ with capacity of 1 mAh cm^−2^. Along with the cycles processing, the overpotential of the cell with pristine Li began to increases at ≈400 h and dropped suddenly at ≈520 h. Normally this sudden overpotential drop is regarded as the sign of short circuit caused by the growth of dendritic Li within the batteries. The longevity of Li^+^‐Zeolite modified Li was extended to ≈1200 h. In stark contrast, the symmetric Li/Li cell with Mg^2+^‐Zeolite layer demonstrated an ultra‐long lifetime of 2100 h and a stable voltage distribution (**Figure** **2**a, red trace, only 55 mV after 2000 h). In addition, the Li dendrites are more likely to generate and further grow under the conditions of high current density or large plating/stripping capacity. So, the current density of 3 mA cm^−2^ (3 mAh cm^−2^) and 2 mA cm^−2^ (6 mAh cm^−2^) were adopted to identify the influences of Mg^2+^‐Zeolite film under the extreme conditions (Figure 2f,i). The symmetric Li/Li cell with pristine Li suffered short circuit due to large dendritic Li after only 160 h. The stabilization time of the cell with Li^+^‐Zeolite layer was elevated to ≈370 h, while that of the cell with Mg^2^‐Zeolite film was further raised to 520 h. Based on the perspective of large deposition capacity (6 mAh cm^−2^), the overpotential of the cell with primitive Li always remained at a steady state with no significant increase before the short circuit. Specifically, there was no kinetic‐limited polarization caused by the thickening of SEI, which explains that the evolution of large Li‐dendrites on the anode surface was the decisive factor for the cell failure. Utilizing only the size‐effect of the channel structure, the Li^+^‐Zeolite layer realized a faster Li^+^ transport at the interface, and thereby a better plating/stripping behavior and the lifetime up to three times. However, a similar situation occurred, that is, a sudden short circuit without the enlargement of overpotential. Obviously, a single means of channels was clearly not sufficient for the protection of lithium metal anodes. Optimizing the deposition behavior of Li on the substrate becomes more critical. The carried Mg^2+^ on the skeleton exerted a further modulating effect, which further elevated the lifetime of Li metal anode to five times. Overall, Mg^2+^‐Zeolite modified Li metal demonstrated the best cyclic stability under the different working conditions, especially at the situation of large deposition capacities.

Mg^2+^‐Zeolite attached Li metal anode possessed the enhanced electrochemical performance, which inevitably corresponds to the change of the electrochemical properties. It is widely recognized that the exchange current density (J_0_) can reflects the speed of kinetic processes within the cells. Compared with pristine Li (J_0_ = 0.22 mA cm^−2^), the J_0_ of lithium foil with Li^+^‐Zeolite (J_0_ = 0.64 mA cm^−2^) and Mg^2+^‐Zeolite (J_0_ = 0.73 mA cm^−2^) were all elevated to different degree, as shown in Tafel curves (Figure 2m; Figure S6, Supporting Information). The carried Mg^2+^ further offered the loading of uniform charge centers and nucleation sites on the surface of lithium metal, which was also consistent with the slightly larger J_0_ of the Li anode with Mg^2+^‐Zeolite layer than that with Li^+^‐Zeolite film. Similarly, CV results of the symmetric Li/Li cell and the asymmetric SS/Li cell all reflected the acceleration of kinetic processes within the cell by the Mg^2+^‐Zeolite layer (Figure 2n,o). The current density of pristine lithium metal rose sharply at ≈0.15 V, which may be related to micro‐short circuit caused by the Li dendrites.

**Figure 3:**
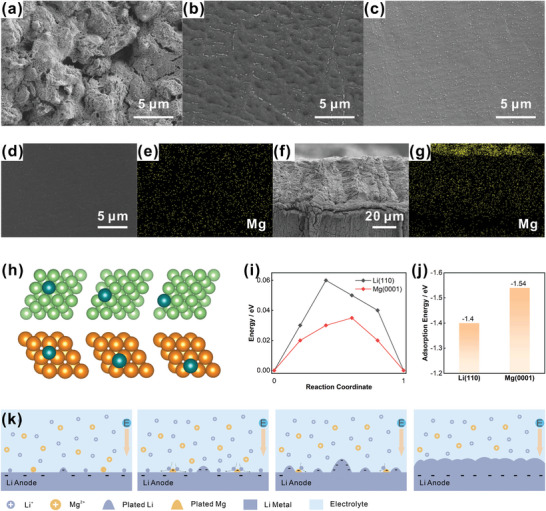
The SEM images of a) pristine Li, b) Li^+^‐Zeolite modified Li, c) Mg^2+^‐Zeolite modified Li surface after plating at 0.5 mA cm^−2^ for 4 h. Element mapping images of Li metal d,e) surface and f,g) cross‐section with Mg^2+^‐Zeolite layer. h) Snapshots of atomic configuration (initial state, transition state and final state, from left to right) along the minimum energy path for the self‐diffusion of Li on Li(110) (top) and Mg(0001) (bottom) in the surface adsorption migration mechanism. i) The corresponding energy along the diffusion paths. j) Adsorption energy of Li on Li(110) and Mg(0001). k) The illustration of protective mechanism of loaded Mg^2+^ for the deposition behavior on the substrate of Li metal.

The service life of lithium metal battery is closely connected with the Li^+^ plating/stripping behavior. To verify the regulatory effect of Mg^2+^‐Zeolite layer on the stability of deposition behavior, the Li metal anode was observed after plating at current density of 0.5 mA cm^−2^ for 4 h via SEM. The deposition situation of pristine Li was poor, which could be reflected by the rough surface with large amounts of filamentous lithium (**Figure** **3**a) and even the large‐scale Li dendrite (Figure S7a, Supporting Information). It can be expected that the dendritic problem is more severe at higher current densities. The needle‐like growth on the surface of Li metal is highly susceptible to result in the SEI rupture (Figure S7b, Supporting Information), the extended contact between Li metal and electrolyte, and the subsequent stronger tendency of the parasitic reactions. During the repeated cycle process, this dendritic lithium further grows into the large dendrite, causing the perforation of separator (Figure S7c, Supporting Information) and the short circuit of battery system. With the size‐effect of channels, the surface state of the lithium anode with Li^+^‐Zeolite layer was greatly ameliorated and exhibits dendrite‐free surface with a corrugated shape. In comparison, the loading of Mg^2+^ on the skeleton exerted a further modulating effect, leading to the optimal plating behavior with the smooth surface (Figure 3c). For working conditions of large current density/capacity, the plating/stripping behavior of Li was similarly well regulated by the Mg^2+^‐Zeolite layer (Figures S8 and S9, Supporting Information). The stable plating/stripping behavior of Li^+^ greatly avoided the formation and growth of Li dendrites on the anode surface, ultimately resulting in the safe and long‐life Li metal anode.

It is well known that the standard reduction potential of Mg^2+^/Mg is higher than that of Li^+^/Li by 0.67 V. Typically, the presence of a reduction potential difference will lead to the chemical reduction of Mg^2+^ to the lithium metal surface until the complete consumption. Therefore, the distribution situation of Mg on the surface and the cross‐section of Li‐metal anode was investigated with EDX technique. Deposited Mg was uniformly distributed over the surface. Similarly, uniform Mg was recognized throughout the cross‐section with a strong signal. Comprehensively analyzing the distribution of loaded ions, it is inferred that the loaded Mg^2+^ was not completely reduced at once on the Li metal surface, but was continuously co‐deposited with Li^+^ during the electro‐treatment process. Furthermore, the element content of Mg on the Mg^2+^‐Zeolite layer reduced only from 5.17% to 5.0% after 100 cycles at a current density of 0.5 mA cm^−2^ (Figures S11 and S12, Supporting Information). It is inferred that the Mg^2+^ carried on the zeolite skeleton probably realized a slow‐release process, which affords a long‐term stabilization effect for Li plating/stripping behavior.

Thermodynamically, lithium metal with high migration energy is more prone to induce low‐dimensional whiskers or dendrites. Lithium metal favors island growth in the early nucleation stage.^[^ ^23^ ^]^ More Li‐ions are preferentially deposited around the island tips rather than on the other smooth regions of the anode due to the higher electric field at the high curvature protrusions. The uneven deposition of Li^+^ at the protrusions gradually evolves to dendritic Li during the continuous electroplating process.^[^ ^24^ ^]^ The deposition structure of a particular metal is closely related to the diffusion process. Specifically, the self‐diffusion path and the corresponding diffusion barrier are key parts of the diffusion kinetics, which then determine the deposition morphology.^[^ ^23^ ^]^ To evaluate the impact of loaded Mg^2+^ on the Li^+^ plating/stripping behavior, the diffusion of lithium atoms on the magnesium/lithium surface were calculated by density functional theory (DFT) method. The most stable surfaces in the Mg metal and the Li metal are Mg(0001) and Li(110), so the adsorption migration paths of lithium atoms and the corresponding energy barriers on these two surfaces were verified. The absorption transport barrier of Li on Li metal surface over the adjacent top sites was 0.06 eV, whereas the migration barrier of Li between the neighboring hcp site on the Mg(0001) was only 0.035 eV (Figure 3h,i; Figure S13, Supporting Information). In comparison to the self‐migration on the bare Li, the diffusion capacity of Li on the Mg surface was boosted. That is, the lithium diffusion rate on the lithium substrate can be significantly enhanced given the presence of deposited Mg. Intriguingly, the adsorption energy of Li on the Mg(0001) was more negative (Figure 3j), which means that lithium atoms will preferentially adsorb and diffuse on the Mg(0001). Combined with the element distribution of Mg on the cross‐section of deposited lithium, it is concluded that the co‐plating of Mg^2+^ carried on the skeleton altered the mode of Li^+^ plating. During the initial Li‐island growth stage, the Mg^2+^ achieved a “competitive growth” plating model that competes with the high curvature region for growth through the enhancement of the fast Li surface diffusion, avoiding the selective deposition at the lithium tip (Figure 3k). Furthermore, the continuous release of Mg also ensured the uniform plating of lithium in the subsequent stages. The regulation of the new deposition model promoted the formation of a smooth surface, which in turn promoted the long‐term stability of the lithium anode.

Typically, Li^+^ undergo five stages throughout the entire deposition process: the movement of Li^+^ solvation sheath in the electrolyte, the desolvation of Li^+^‐solvated group at the interface, the diffusion of bare Li^+^ through the SEI, the plating of Li^+^ (Li^+^ + e^−^ → Li), and the migration of Li atoms on the Li metal substrate.^[^ ^25^ ^]^ In the EIS spectra, the results of these related kinetic processes acting together are only presented over a limited bandwidth. The feature peaks are highly overlapping and cannot be easily separated, leading to the ambiguity and uncertainty in the diagnostic analysis of EIS.^[^ ^26^ ^]^ The distribution relaxation time (DRT) is an efficient method to separate, resolve, and quantify the highly overlapping electrochemical processes within lithium‐ion batteries. The relaxation time is the time required for the variables to converge from transient state to steady state in a system. The different electrochemical processes correspond to the different relaxation times.^[^ ^26^ ^]^ Therefore, the various electrochemical processes can be recognized and understood based on the information of the characteristic peaks in the DRT function. Typically, the relaxation time τ of symmetric Li/Li cell with liquid electrolyte is concentrated in the range of 10^−5^–10 s, which is basically divided into two parts: the irreversible part (10^−5^–10^−4^ s) and the reversible part (10^−2^–10 s).^[^ ^27^ ^]^ The characteristic peaks in the time range of 10^−5^–10^−4^ s are related to the Li^+^ transport through the solid electrolyte interface, called as R_int_. Small changes of SEI properties can be reflected in R_int_. Relaxation peaks located at 10^−2^–10 s are initiated by the charge transfer process of Li^+^ and the migration of lithium atoms on the substrate, i.e., R_ct_ and R_d_.^[^ ^28^ ^]^ The time‐domain‐based DRT is realized by deconvolution of EIS spectrum.^[^ ^29^ ^]^ For the insight of the dynamic evolution on the surface of Li metal anode, the symmetric Li/Li cell with and without Mg^2+^‐Zeolite at various cycling phases were monitored with the assistance of EIS‐DRT method.

**Figure 4:**
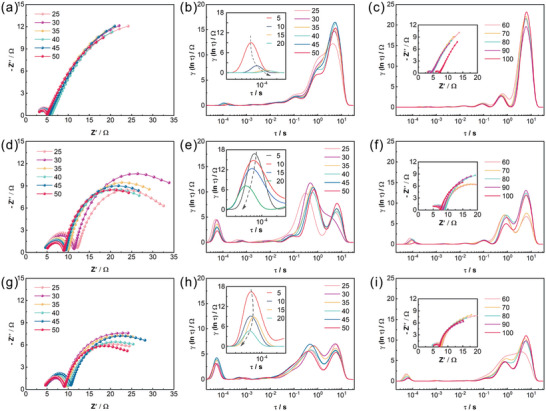
The evolution of interfacial states for symmetric Li/Li cells. For symmetric Li/Li cell with pristine Li: a) the EIS spectra from 25 to 50 cycles, b) the corresponding DRT spectra and insert DRT spectra before 20 cycles, c) the DRT spectra from 60 to 100 cycles and insert the corresponding EIS spectra. For symmetric Li/Li cell with Li^+^‐Zeolite layer: d) the EIS spectra from 25 to 50 cycles, e) the corresponding DRT spectra and insert DRT spectra before 20 cycles, f) the DRT spectra from 60 to 100 cycles and insert the corresponding EIS spectra. For symmetric Li/Li cell with Mg^+^‐Zeolite layer: g) the EIS spectra from 25 to 50 cycles, h) the corresponding DRT spectra and insert DRT spectra before 20 cycles, i) the DRT spectra from 60 to 100 cycles and insert the corresponding EIS spectra.

With the charge/discharge cycling, the R_int_ of cell with pristine Li foil decreased drastically to a negligible value (**Figure** **4**b). This phenomenon does not imply the stabilization of SEI properties and the decrease of impedance. Instead, it was caused by the irreversible micro‐short circuit within the cell, which signified the fundamental change of charge transport mechanism.^[^ ^30^ ^]^ The R_int_ is sensitive to the tiny change of SEI state. Typically, Li^+^ migrate across the SEI and deposit at SEI/Li interface. However, deposited lithium tends to grow in 1D dendritic form, which easily punctures the SEI, thereby inducing the deposition behavior of Li^+^ at the new Li/electrolyte interface. In this case, the deposition location of lithium changes from SEI/Li interface to Li/electrolyte interface, which is also reflected as the decreases of R_int_ in EIS spectrum. In addition, the peak of R_int_ shifted to a higher time constant. The Li^+^ transport response through the interface became slower, that is, the Li^+^ transport capacity of SEI derived on the surface of pristine Li foil consistently declined. In terms of intrinsic property of SEI, the low‐coordinated solvation groups within the channel of zeolite induced an anion‐derived SEI, which is more stable and robust than nature SEI on the surface of bare Li. With the evolution and stabilization of SEI, the R_int_ peak of cell with Li^+^‐Zeolite layer showed a decreasing trend. More importantly, it gradually moved toward the lower time constant, i.e., the faster Li^+^ interfacial transport response (Figure 4e). Li^+^‐Zeolite film remarkably contributed to the formation of stable SEI with higher Li^+^ transport capacity by the size‐effect of the channels. As well, the symmetric Li/Li cell with Mg^2+^‐Zeolite had a similar R_int_ peak shift state (Figure 4h). The subsequent charge transfer of Li^+^ and migration of Li atoms on Li substrate involves time constants ≈0.2 and 5 Hz, respectively. With cycling, the R_ct_ of cell with pristine Li foil displayed a declining behavior (Figure 4b), which was attributed to the same reason for the decrease of R_int_. This was also corroborated by the continuous deterioration of Li metal surface, namely the increase of migration impedance (R_d_) for Li‐atoms on the substrate. Compared to the cell with Li^+^‐Zeolite layer, the cell with Mg^2+^‐Zeolite film exhibited a stable and minimal R_ct_, which was related with the loading of charging centers and nucleation sites caused by loaded Mg^2+^. Likewise, Mg^2+^‐Zeolite modified Li anode with high diffusion ability always delivered the smallest R_d_. From the DRT results, it can be concluded that both the transport kinetics through the interface and the deposition behavior on the lithium substrate were all improved by the Mg^2+^‐Zeolite layer. The symmetric Li/Li cell with pristine Li delivered a continuous deterioration tendency with operating time, whereas the cell with Mg^2+^‐Zeolite maintained a stable R_int_, R_ct_, and R_d_ under minor fluctuations (Figure 4c,i). Overall, all these results illustrated the high efficiency of Mg^2+^‐Zeolite layer in enhancing the plating/stripping stability of Li metal.

**Figure 5:**
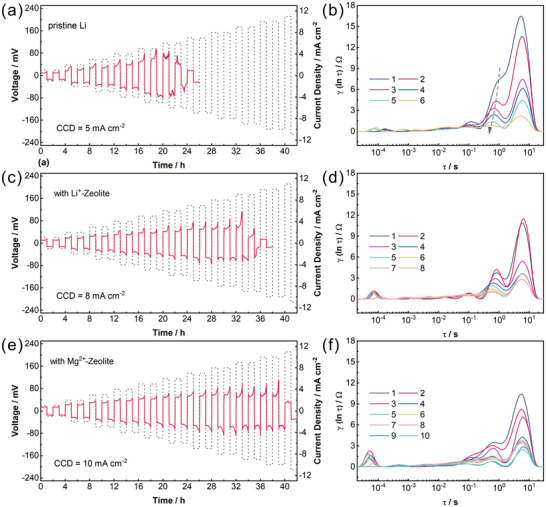
The investigation of critical current density and the evolution of interfacial states for symmetric Li/Li cell. a) Voltage profiles and b) DRT spectra for cell with pristine Li. c) Voltage profiles and d) DRT spectra for cell with Li^+^‐Zeolite layer. e) Voltage profiles and f) DRT spectra for cell with Mg^+^‐Zeolite layer.

The maximum tolerated current density of lithium‐ion batteries with no failure is normally defined as the critical current density (CCD). Theoretically, the intrinsic properties of interface chemistry within the cell can be reflected by the CCD, which is jointly determined by the maximum ion transport flux per unit area/time and the charge transfer rate at interface.^[^ ^31^ ^]^ For the Li metal anodes, CCD also reflects the ability of the material matrix to resist the dendritic growth at the high currents. CCD of the symmetric Li/Li cell with and without Mg^2+^‐Zeolite layer were characterized with the incremental current densities, followed by the DRT analysis for the interface of the cell. The current density was increased from 1 mA cm^−2^ in steps of 1 mA cm^−2^ with the plating/stripping time of 1 h. To avoid the impact of metastable SEI, the CCD of the cell was evaluated after 80 cycles at current density of 1 mA cm^−2^ (1 mAh cm^−2^). The pristine Li‐assembled cell exhibited a CCD of only 5 mA cm^−2^ with an abrupt signal of short circuit at 6 mA cm^−2^ (**Figure** **5**a). By comparison, the CCD of cell with Li^+^‐Zeolite layer increased to 8 mA cm^−2^ (Figure 5c), which signifies the superiority of the channel structure for the modulation of the Li^+^ plating/stripping behavior. Due to the regulation of depositional model, the Mg^2+^ loaded on the zeolite backbone further boosted the critical current density to 10 mA cm^−2^ (Figure 5e). As the current rises, the probability of dendritic Li growing and further piercing the SEI strengthens incrementally. Lithium dendrites penetrating SEI may alter the deposition mode of Li^+^. Instead of diffusing through the SEI to the surface of lithium anode, part of Li ions tend to get electrons and deposit on the portion of dendritic Li that grows out of SEI.^[^ ^32^ ^]^ As the current density going up from 1 mA cm^−2^ to 6 mA cm^−2^, the R_int_/R_ct_/R_d_ of cell with blank Li all displayed a cliff‐like drop (Figure 5b), which also means the sharp deterioration of the lithium metal surface state. The kinetic peaks of cell with Mg^2+^‐Zeolite layer demonstrated only a slight downward trend (Figure 5f), and this advantage is more pronounced at high currents density.

**Figure 6:**
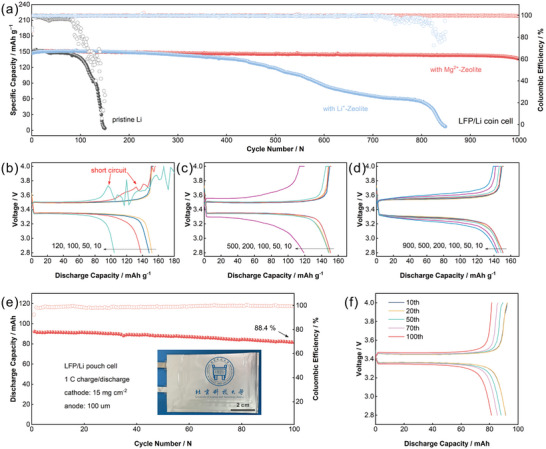
a) The cyclic stability and the corresponding charge/discharge profiles of LiFePO_4_/Li coin cell with b) pristine Li, c) Li^+^‐Zeolite modified Li and d) Mg^2+^‐Zeolite layer modified Li. e) The cyclic stability and f) the corresponding charge/discharge profiles of LiFePO_4_/Li pouch cell with Mg^2+^‐Zeolite layer modified Li at a current density of 1 C.

The protective effectiveness of the Mg^2+^‐Zeolite layer for Li metal anode was finally confirmed in the LiFePO_4_/Li (LFP/Li) cell. It is well known that DOL/DME has good applicability with lithium metal anodes. Meanwhile, LiFePO_4_ is a stable cathode material with excellent cycling stability. Therefore, the cyclic stability of the LFP/Li cell is mainly influenced by the properties of lithium metal anode. The initial specific capacities of LFP/Li coin cells assembled with pristine Li, Li^+^/Mg^2+^‐Zeolite modified lithium were all in the range of 145–150 mAh g^−1^ (**Figure** **6**a), and are not obviously different. Apparently, the cell with pristine Li was the most unstable, with the complete failure at <150 charge/discharge cycles. Continuously decreasing Coulombic efficiency means constant deterioration at the interface of lithium metal anode, which is mainly attributed to the growth of dendritic Li, the subsequent formation of dead lithium and the accompanying side reactions. At 100 cycles, an unexpected drop of voltage can be observed in the charge curve (Figure 6b), revealing the micro‐short circuit caused by lithium dendrites within the cell. In the ensuing charging process, the dendritic Li was fractured into electrically inactive “dead lithium”, which is reflected in the voltage return. The generation and fracture of lithium dendrites were shown as the voltage fluctuations in the charge curves. Accompanied with the dramatic drop of discharge capacity, this condition of micro‐short circuit was more severe within the lithium metal cell after 120 cycles. Likewise, the surge of interface impedance exhibited the poor situation of the lithium surface, as shown in the Nyquist curve of the cell assembled with pristine Li (Figure S14, Supporting Information). The cell with Mg^2+^‐Zeolite layer still showed a discharge specific capacity retention of 91% and a coulombic efficiency over 99.5% after 1000 cycles at a rate of 1 C. As well, the stable interfacial properties further confirmed the superiority of the Mg^2+^‐Zeolite layer in ensuring the cyclic stability of lithium metal anode.

The pouch cells, using a high‐loading LiFePO_4_ cathode (15 mg cm^−2^) and a 100 µm thick lithium metal anode, were assembled and evaluated to demonstrate the practical effectiveness of Mg^2+^‐Zeolite layer. Under the severe current density of 1 C charge/1 C discharge, the Li metal pouch cell with Mg^2+^‐Zeolite film maintained a capacity retention of 88.4% after 100 cycles (Figure 6e). In addition, the stable coulombic efficiency also reflected a secure working state and a great Li deposition behavior within the cell. In the same situation, the pouch cell with pristine Li remained in a steady state for only five cycles and then suffered a dramatic loss of capacity (Figure S16, Supporting Information). On the other hand, the pouch cell realized a capacity retention of 94.8% after 180 cycles at 0.5 C charge/discharge (Figure S17, Supporting Information), which confirms the dependability of the Mg^2+^‐Zeolite layer at different operating conditions. Overall, the synergistic Mg^2+^‐Zeolite layer from both the acceleration of Li transport kinetics and the regulation of Li^+^ deposition pattern efficiently protects the lithium metal anode from the hazards of Li dendrite, which may be a promising way to realize the safe and long‐life lithium metal batteries.

## 3 Conclusion

In conclusion, the lithium metal protective layer constructed by Mg^2+^‐Zeolite simultaneously achieves the fast ion transport kinetics at the interface and the regulation of Li deposition model on the lithium substrate, thereby facilitating the dendrite‐free deposition. The channels within the protective layer provide the low‐coordinated Li^+^‐solvated group since the size‐effect, leading to the faster desolvation process and the stable, ion‐conductive SEI. In addition to the loading of uniform charge centers and nucleation site, the co‐deposition of sustained release Mg^2+^ gives a new faster pathway for the diffusion of Li, which remarkably promotes the uniform Li diffusion on the lithium substrate. With this synergistic protective mechanism, the Mg^2+^‐Zeolite modified Li can stably cycle more than 2100 h at a current density of 1 mA cm^−2^. In addition, the LiFePO_4_/Li pouch cell realizes a capacity retention of 88.4% after 100 cycles at a severe current density of 1 C. It is expected that this simple and efficient strategy gives new guidance for the practical applications of long‐life lithium metal anodes.

## 4 Experimental Section

### Preparation of Mg^2+^‐Zeolite Layer

The 3A zeolite molecular sieve was purchased from Alfa Aesar. The 3A zeolite molecular sieve powder was fully stirred in 0.2 m LiOH solution at 80 °C for 12 h to accomplish the exchange of cations and Li^+^ in zeolite backbone. The collected powder was then washed three times with deionized water to completely remove residual LiOH. After that, the powder was calcined at 400 °C for 5 h to remove water from zeolite pores to obtain Li^+^‐loaded zeolite molecular sieve, remarked as Li^+^‐Zeolite. The Li^+^‐Zeolite powder was fully stirred in 1 m MgCl_2_ solution at 80 °C for 24 h. Then the above steps were repeated to prepare Mg^2+^‐loaded zeolite molecular sieve (remarked as Mg^2+^‐Zeolite). The content of Mg in Mg^2+^‐Zeolite was measured as 5.18 wt.% by inductively coupled plasma‐optical emission spectroscopy (ICP‐OES). Li^+^/Mg^2+^‐Zeolite powder was mixed thoroughly with PVDF in an 8:1 ratio in an appropriate amount of NMP. The prepared slurry was coated on glass plate and dried at 80 °C in vacuum for 12 h until the NMP was completely volatilized. After complete volatilization of NMP, the Li^+^/Mg^2+^‐Zeolite film was soaked in methanol for 5 min and peeled off from the glass plate. The prepared film was cut into small discs (12 mm in diameter) and compacted with physical pressure, and subsequently transferred inside a glove box (H_2_O/O_2_ < 1 ppm) for future use. The thickness of Li^+^/Mg^2+^‐Zeolite layer is ≈25 µm.

### The Preparation of Coin Cells and Pouch Cells

The fabricated Li^+^/Mg^2+^‐Zeolite films were tightly attached to Li metal anode through the physical pressing. Ceglard 2400 was used as separator and 1 m LiFSI‐DOL/DME was used as electrolyte. Symmetric Li/Li coin cells and LiFePO_4_/Li coin cells (CR2025 type) were assembled in an argon‐filled glove box (H_2_O/O_2_ < 0.1 ppm). LiFePO_4_/Li pouch cells were assembled in a dry room at a dew point of −60 °C. The cathode is composed of Al current collector and double‐layer active materials. In addition, the mass loading of cathode is 15 mg cm^−2^, and the thickness of lithium foil is 100 µm. First, the nickel current collector was connected to the lithium foil anode. Second, the cathodes and anodes were stacked layer‐by‐layer with separator. Third, aluminium cell tabs were welded to the Al current collector by ultrasonic spot welding. Fourth, it was packed by aluminium plastic film. Finally, the electrolyte was injected into the pouch cell, and the pouch cell was sealed.

### Electrochemical Characterizations

Symmetric Li/Li cells were used to assess Li plating/stripping behavior on the surface of anode at current density of 1 mA cm^−2^ (plating capacity of 1 mAh cm^−2^), 2 mA cm^−2^ (plating capacity of 6 mAh cm^−2^) and 3 mA cm^−2^ (plating capacity of 3 mAh cm^−2^), respectively. Critical current density test was conducted with incremental current densities from 1 mA cm^−2^ to 12 mA cm^−2^ with step size of 1 mA cm^−2^ and plating/stripping time of 1 h. LiFePO_4_/Li cion cells and LiFePO_4_/Li pouch cells were monitored in voltage range of 2.8–4 V to assess the effect of Li^+^/Mg^2+^‐Zeolite layer on cyclic stability of Lithium anode in Li metal battery system. All cells were evaluated at room temperature via LAND battery test system (CT3001A). Cyclic voltammetry (CV) and Tafel tests were measured on an electrochemical workstation (VersaATAT3) to monitor the Li^+^ plating/stripping behavior. With scan rate of 0.1 mV s^−1^, the CV tests of asymmetric stainless steel (SS)/Li cell and symmetric Li/Li cell were performed in voltage range of −0.1–1 V and −0.2–0.2 V, respectively. The exchange current density (J_0_) for Li foil without and with M‐Zeolite layer was calculated by linear fitting of Tafel curves, which operated with scan rate of 0.1 mV s^−1^ and voltage range of −0.1–0.1 V. The EIS of symmetric Li/Li cell and LiFePO_4_/Li full cell were carried out in the frequency range of 0.1–10^5^ Hz.

### Characterization

After electrochemical treatment, symmetric Li/Li cell was disassembled carefully to get cycled Li metal anode. Then, Li metal sheet was rinsed three times repeatedly by dimethoxyethane (DME, Macklin, 99%) to wash away electrolyte salt and residual solvent on surface, and dried in vacuum for 6 h for subsequent characterization. Scanning electron microscope (SEM, ZEISS Gemini 300) was utilized to observe the plating/stripping morphology of Li^+^. The EDX technique was also employed to investigate the elemental distribution of Mg on Li substrate. X‐ray photoelectron spectroscopy (XPS, Thermo Scientific K‐Alpha) with Al Kα‐rays (1486.6 eV) was exploited to analyze the composition of SEI on Li metal surface. Mg^2+^‐Zeolite plates were immersed into 1 m LiFSI‐DOL/DME under 70 °C for 48 h to soak electrolyte molecules into Mg^2+^‐Zeolite channels. The plates were then wiped and dried at 60 °C for 24 h under vacuum to get rid of electrolyte solvents on the surface. Fourier transform infrared spectroscopy (FTIR, Thermo Scientific Nicolet iS20) and Raman spectroscopy (Raman, HORIBA Scientific LabRAM HR Evolution) were conducted to explore Li^+^‐solvated structure inside M‐Zeolite channels.

### Calculation Details

In the work, the Vienna Ab initio Simulations Package (VASP)^[^ ^33^ ^]^ was utilized to carried out all DFT calculations. The ion‐electron interaction and the correlation‐exchange interaction were described through employing the projector‐augmented wave method (PAW) and the generalized gradient approximation (GGA) with the Perdew–Burke–Ernzerhof (PBE) density functional respectively.^[^ ^34^ ^]^ A plane wave basis set with a kinetic energy cutoff of 500 eV. The electronic energy was considered self‐consistent when the energy change was <10^−5^ eV. A geometry optimization was considered convergent when the energy change was smaller than 0.03 eV Å^−1^. For the surface models, Six layers were built for all structures where the bottom half layers are fixed to mimic the bulk structure with the top half layers fully relaxed where a 3×3×1 Gamma‐center k‐point sampling was used. In addition, the climbing image nudge elastic band (CI‐NEB) and Dimer method were employed^[^ ^35^ ^]^ to study the transition states. For the transition states, four images were inserted along the minimum energy path (MEP) to calculate the saddle points and the simulations would be stopped until the forces were <0.03 eV Å^−1^.

## Conflict of Interest

The authors declare no conflict of interest.

## Supporting information

Supporting Information

## Data Availability

The data that support the findings of this study are available from the corresponding author upon reasonable request.
