# Supplementary material for: Cation‐Loaded Porous Mg2+‐Zeolite Layer Direct Dendrite‐Free Deposition toward Long‐Life Lithium Metal Anodes
Source: Adv Sci (Weinh). 2024 Apr 10;11(23):2308939. doi: 10.1002/advs.202308939 (PMC11187884; doi:10.1002/advs.202308939)
Supplement: Supplementary file 1 — Supporting Information [file ADVS-11-2308939-s001.pdf]

ADVANCED SCIENCE
Open Access

Supporting Information

for *Adv. Sci.*, DOI 10.1002/advs.202308939

Cation-Loaded Porous Mg^{2+}-Zeolite Layer Direct Dendrite-Free Deposition toward Long-Life Lithium Metal Anodes

Ben Su, Xingyu Wang, Lei Chai, Sida Huo, Jingyi Qiu, Qiang Huang, Shuang Li, Yue Wang and Wendong Xue**

Cation-Loaded Porous Mg^{2+}-Zeolite Layer Direct Dendrite-Free Deposition Towards Long-Life Lithium Metal Anodes

Ben Su[1], Xingyu Wang[1], Lei Chai[3], Sida Huo[1], Jingyi Qiu[2], Qiang Huang[2], Shuang Li[4], Yue Wang[2], Wendong Xue[1]**

Ben Su, Xingyu Wang, Sida Huo, Wendong Xue
Address line 1: School of Materials Science and Engineering, University of Science and Technology, Beijing, Beijing 100083, China
Jingyi Qiu, Qiang Huang, Yue Wang
Address line 2: Research Institute of Chemical Defense, Beijing, Beijing 100191, China
Lei Chai
Address line 3: School of Microelectronics, Dalian University of Technology, Dalian, Liaoning 116024, China
Shuang Li
Address line 4: School of Materials Science and Engineering, Nanjing University of Science and Technology, Nanjing, Jiangsu 210094, China

E-mail: xuewendong@ustb.edu.cn; wangyuegt@163.com

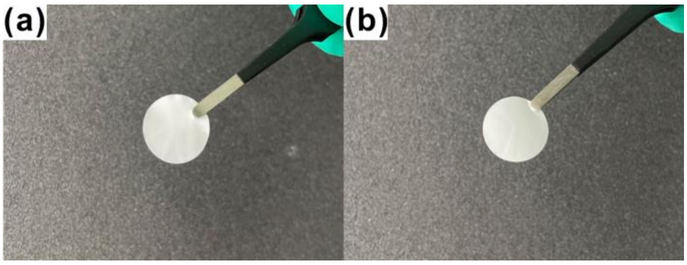

Figure S1. The Photographs of (a) Li^{+}-Zeolite layer and (b) Mg^{2+}-Zeolite layer.

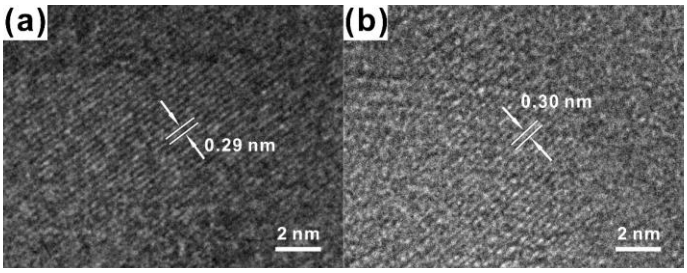

Figure S2. The channel structure of (a) Li^{+}-Zeolite and (b) Mg^{2+}-Zeolite.

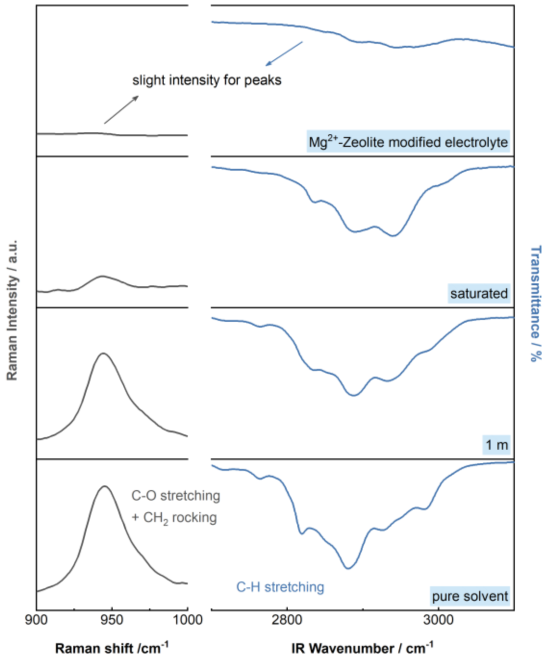

Figure S3. The Raman and FTIR spectra for various electrolyte.

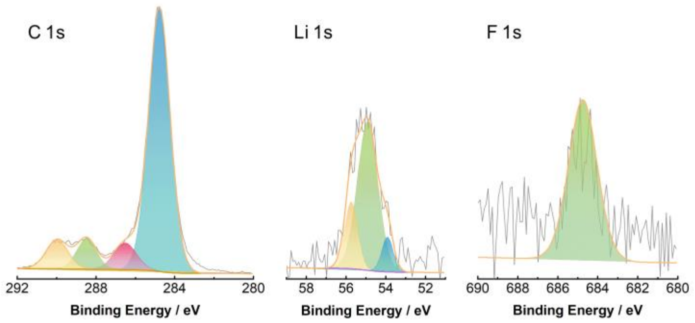

Figure S4. XPS spectra of Li^{2+}-Zeolite layer modified Li after 10 h of cycling.

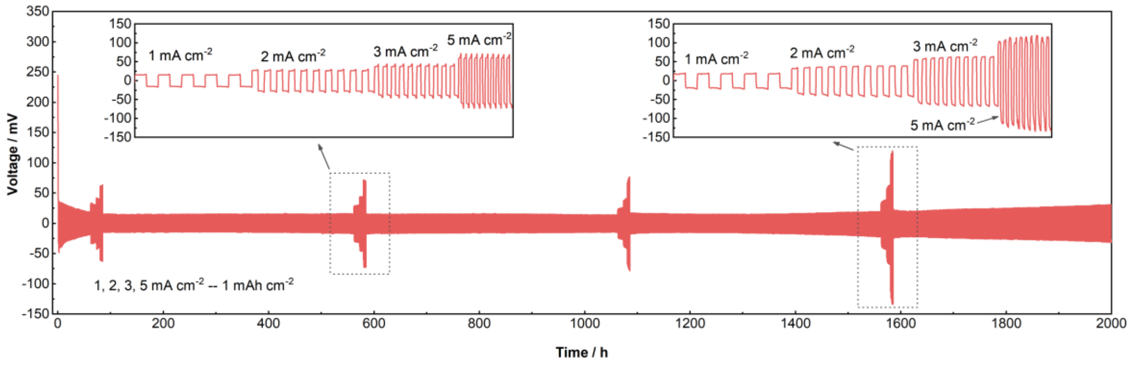

Figure S5. The Li plating/stripping voltage curves of symmetrical Li/Li cells with Mg^{2+}-Zeolite at the current density of 1, 2, 3, 5, 1 mA cm^{-2} with a plating/stripping capacity of 1 mAh cm^{-2}.

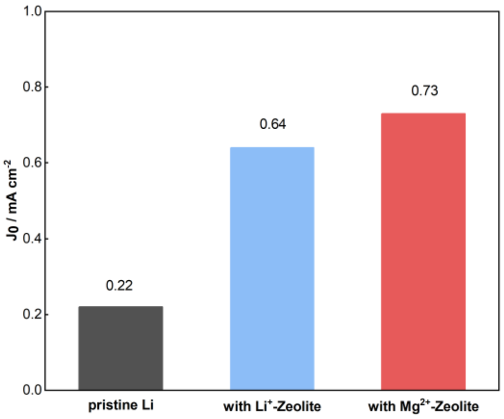

Figure S6. The exchange current density (J_0) calculated from the Tafel plots in Fig. 2m.

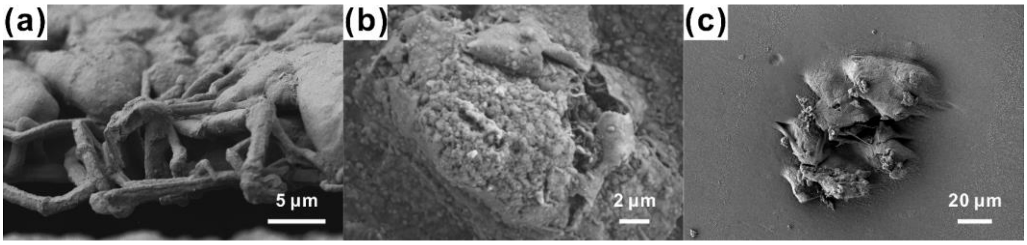

Figure S7. The SEM image of plated Li on the surface of pristine Li: (a) cross section, (b) surface. (c) Separator puncture caused by dendritic Li formatted on the pristine Li anode.

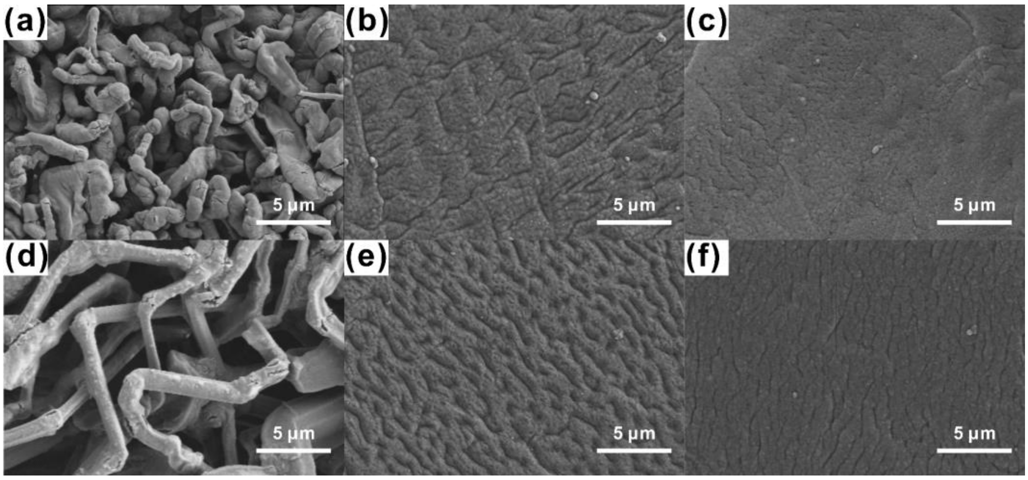

Figure S8. The SEM images of (a, d) pristine Li, (b, e) Li^+-Zeolite modified Li, (c, f) Mg^{2+}-Zeolite modified Li surface after plating at 4 mA cm^{-2} for 2 mAh cm^{-2} and 0.5 mA cm^{-2} for 4 mAh cm^{-2}, respectively.

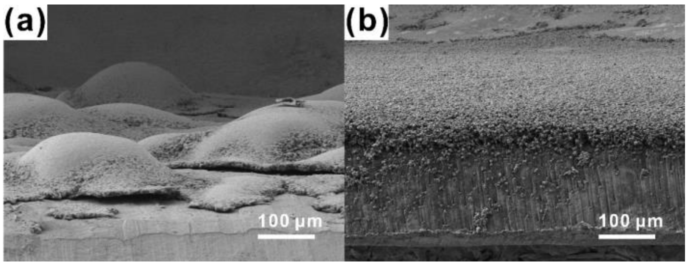

Figure S9. The SEM image of plated Li anode cross-section without and with Mg^{2+}-Zeolite layer.

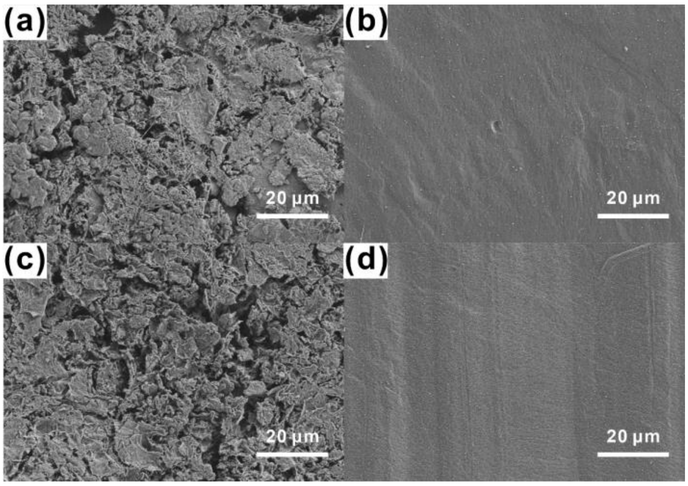

Figure S10. The SEM images of (a, c) pristine Li surface and (b, d) Mg^{2+}-Zeolite modified Li surface after 20 cycles and 50 cycles at 0.5 mA cm^{-2} (with capacity of 0.5 mAh cm^{-2}), respectively.

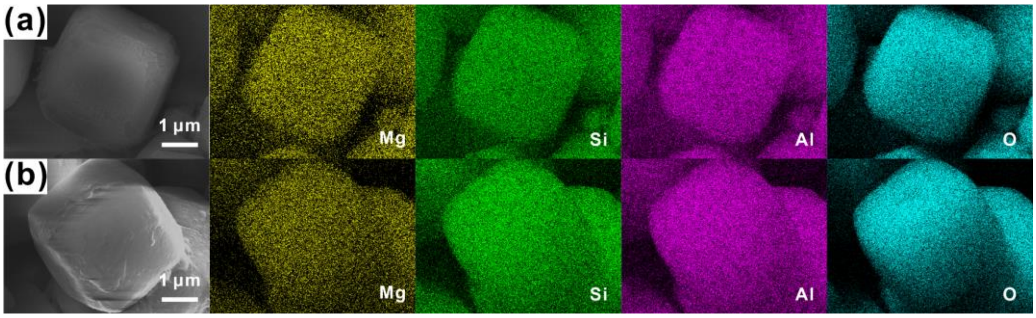

Figure S11. Element mapping images of Mg^{2+}-Zeolite before and after 100 plating/stripping cycles.

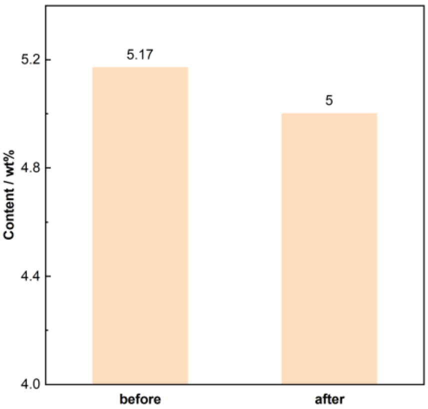

Figure S12. Element content of Mg on the Mg^{2+}-Zeolite before and after 100 plating/stripping cycles.

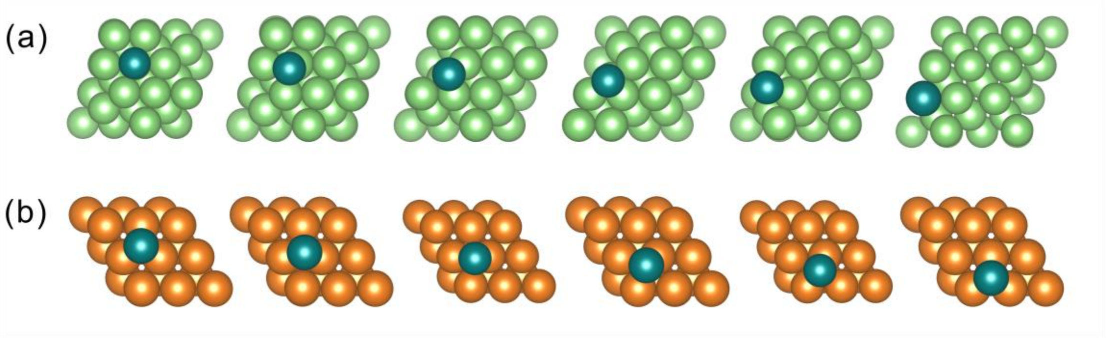

Figure S13. Snapshots of atomic configuration (from left to right) along the minimum energy path for the self-diffusion of Li on (a) Li(110) and (b) Mg(0001) in the surface adsorption migration mechanism.

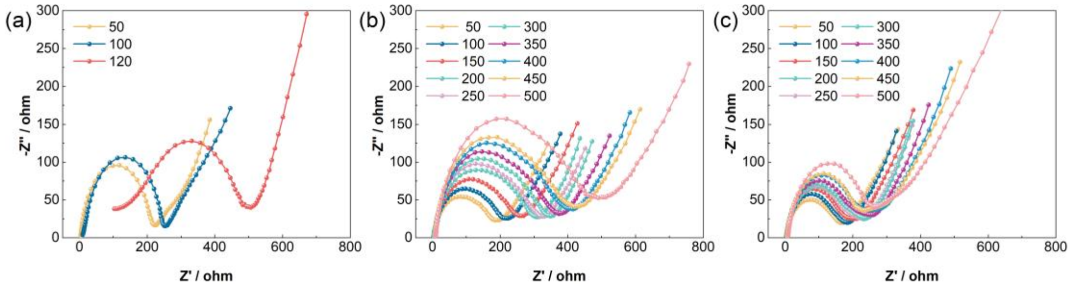

Figure S14. The EIS curves of $LiFePO_4$/Li coin cell with (a) pristine Li, (b) Li^+-Zeolite modified Li and (c) Mg^{2+}-Zeolite layer modified Li.

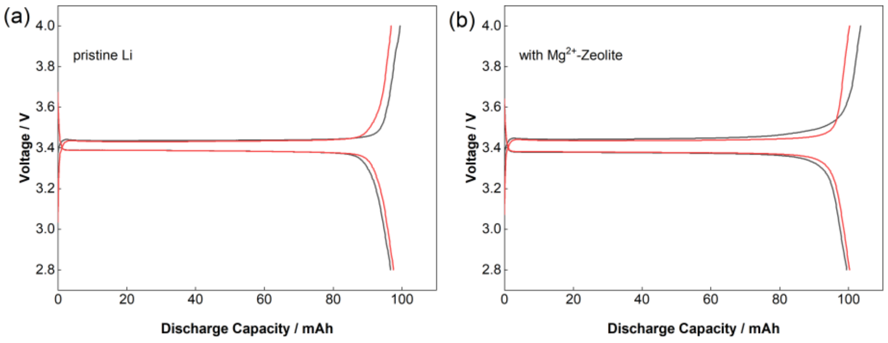

Figure S15. The 1st and the 2nd charge/discharge curves of $LiFePO_4$/Li pouch cell with (a) pristine Li and (b) Mg^{2+}-Zeolite modified Li.

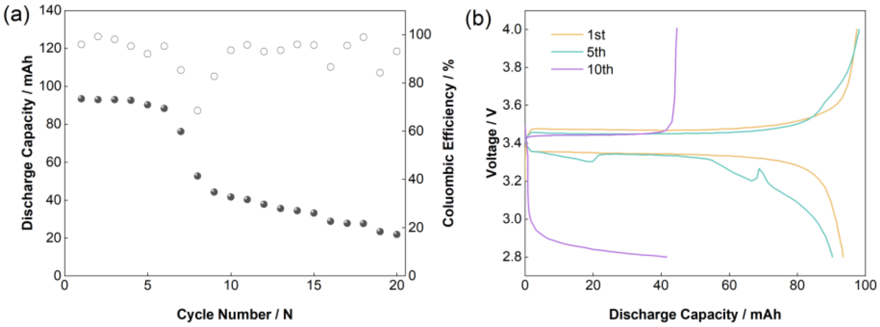

Figure S16. (a) The cyclic stability and (b) the corresponding charge/discharge curves of $LiFePO_4$/Li pouch cell with pristine Li at a current density of 1 C.

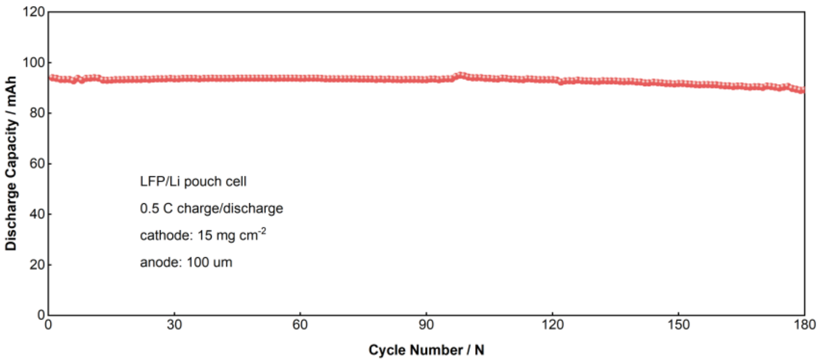

Figure S17. The cyclic stability of $LiFePO_4$/Li pouch cell with Mg^{2+}-Zeolite layer modified Li at a current density of 0.5 C.
